# Interpreting response time effects in functional imaging studies

**DOI:** 10.1016/j.neuroimage.2014.05.073

**Published:** 2014-10-01

**Authors:** J.S.H. Taylor, Kathleen Rastle, Matthew H. Davis

**Affiliations:** a Department of Psychology, Royal Holloway University of London, Egham Hill, Egham TW20 0EX, UK; b Medical Research Council Cognition and Brain Sciences Unit, 15 Chaucer Road, Cambridge CB2 7EF, UK

**Keywords:** Response time, Reading aloud, Neuroimaging, Regularity, Lexicality, Learning

## Abstract

It has been suggested that differential neural activity in imaging studies is most informative if it is independent of response time (RT) differences. However, others view RT as a behavioural index of key cognitive processes, which is likely linked to underlying neural activity. Here, we reconcile these views using the effort and engagement framework developed by Taylor, Rastle, and Davis (2013) and data from the domain of reading aloud. We propose that differences in neural *engagement* should be independent of RT, whereas, differences in neural *effort* should co-vary with RT. We illustrate these different mechanisms using data from an fMRI study of neural activity during reading aloud of regular words, irregular words, and pseudowords. In line with our proposals, activation revealed by contrasts designed to tap differences in neural engagement (e.g., words are meaningful and therefore engage semantic representations more than pseudowords) survived correction for RT, whereas activation for contrasts designed to tap differences in neural effort (e.g., it is more difficult to generate the pronunciation of pseudowords than words) correlated with RT. However, even for contrasts designed to tap neural effort, activity remained after factoring out the RT–BOLD response correlation. This may reveal unpredicted differences in neural engagement (e.g., learning phonological forms for pseudowords > words) that could further the development of cognitive models of reading aloud. Our framework provides a theoretically well-grounded and easily implemented method for analysing and interpreting RT effects in neuroimaging studies of cognitive processes.

## Highlights

• We propose a way to interpret correlations between activity and RT in fMRI studies.
• Activity for contrasts tapping neural engagement should be independent of RT.
• Activity for contrasts tapping neural effort should correlate with RT.
• RT correlated activity during pseudo and irregular word naming supports our proposal.
• Additional engagement for pseudo and irregular words requires further investigation.

## Introduction

A key experimental method in both cognitive psychology and cognitive neuroscience involves asking participants to perform specific tasks on selected stimuli and collecting behavioural (accuracy, response time) and/or haemodynamic outcome measures. Statistical comparisons of these measures allow researchers to draw increasingly specific inferences concerning the underlying cognitive and neural processes that contribute to task performance.

However, despite this similarity in approach, psychologists and neuroscientists often differ in their treatment of a behavioural outcome measure – response time (RT) – that is routinely collected in these experiments. Neuroscientists have sometimes argued that RT differences confound comparisons of brain activity between conditions, and have thus employed a variety of approaches to exclude these apparently ‘uninteresting’ RT-associated neural responses (Binder et al., 2005, Christoff et al., 2001, Crittenden and Duncan, 2012, Graves et al., 2010) or used passive perception designs to minimise the influence of task performance (Ben-Shachar et al., 2011, Pulvermüller et al., 2012, Vinckier et al., 2007, Wright et al., 2011). In contrast, since the time of Donders (1969/1868), behavioural studies have used RT as a key dependent measure to support the inference that different types of stimuli are represented and/or processed in different ways.

In this paper we propose a framework to explain which between-condition differences in neural activity should be independent of RT. We then set out a method for both regressing out and including RT-associated variance when analysing functional magnetic resonance imaging (fMRI) data. We demonstrate the effectiveness of this approach in analysing neuroimaging data collected during reading aloud.

### Response time effects in brain imaging and behavioural studies

Evoked haemodynamic responses often increase with the duration of stimulation (Boynton et al., 1996, Horner and Andrews, 2009), and hence should also increase with the time spent on task. This observation has led to concerns regarding the appropriate treatment of neuroimaging contrasts between conditions that differ in RT. The nature of the concern is that two conditions may produce differential activation not because of a qualitative difference in their underlying neural mechanisms, but because stimulus processing in one condition takes longer than that in the other. Researchers have approached this potential problem in a variety of ways. For example, Crittenden and Duncan (2012) explicitly modelled event duration (RT), allowing them to examine multiple demand network (fronto-parietal cortices) activity under various manipulations of task difficulty, independent of RT. Taking a different approach, Yarkoni et al. (2009) included trial-by-trial RT as a parametric modulator and found that activity in frontal and parietal cortices was positively correlated with RT across several different tasks (working memory, emotional processing, decision making). They suggested that, “RT variability may explain a considerable amount of variance in frontal activation in most tasks” and that this may account for “fMRI effects previously attributed to qualitative differences between experimental conditions” (p. e2457). Yet a different method was used by Binder et al. (2005); a conjunction analysis revealed brain regions in which activity correlated with RT *for all item types* during reading aloud of regular words, irregular words, and pseudowords. It was proposed that RT correlated brain activity *within stimulus type* must arise from “domain general” processing demands. Activation differences *between stimulus types* were therefore only regarded as interesting if they occurred outside of these domain general brain regions. A similar interpretation, although a different method of modelling RT, was applied by Graves et al. (2010) who included multiple psycholinguistic variables, along with RT, as parametric modulators in their analysis of neural activity in an fMRI study of reading. The authors argued that effects of the psycholinguistic variables were of greatest interest if they occurred in areas that did not show positive correlations with RT. Thus, in all these discussed cases it is assumed that differential neural activity only provides evidence of neural specialisation if activation differences cannot be explained by differences in RT.

However, these approaches overlook the information provided by RT variation in behavioural studies. For example, in the Stroop task, patients suffering from psychological disorders are typically slower to name ink colours for words relevant to their clinical condition (Williams et al., 1996), and in the Implicit Association Test, white participants are typically slower to classify black faces and positive words with the same key press than they are to classify black faces and negative words with the same key press (Phelps et al., 2000). In both of these cases, RT differences between conditions indicate underlying processing differences, and we would thus expect differences in neural activity *in regions relevant to performing the task* to correlate with these RT effects, as explicitly demonstrated by Phelps et al. (2000) for the amygdala.

This was acknowledged by Wilson et al. (2009) in their interpretation of neural activity during picture naming. They argued that where RT effects occurred in brain regions in which activity was sensitive to psycholinguistic variables of interest (such as word frequency and concept familiarity) these brain regions were “presumably involved in the stages of word production identified by the other variables in question”. However, RT effects outside of these regions were taken to reflect executive and attentional processes. Whilst this seems sensible, the psycholinguistic variables considered were by no means exhaustive, RT could simply be functioning as a proxy for variables directly relevant to picture naming, but not included in the model, for example, initial phoneme, age-of-acquisition. Similar concerns were raised by Henson (2005) who stated that, as behavioural data (such as RT) and neuroimaging data are both dependent variables, one cannot cause the other. Instead, both are better thought of as different indices of underlying cognitive processes. This was in fact the approach taken in two later studies by Wilson et al., 2010, Wilson et al., 2014. RT was used as a proxy for syntactic complexity when examining activity in inferior frontal gyrus and anterior temporal lobe during syntactic processing in neuropsychological patients.

### The effort and engagement framework

We argue that separating informative from non-informative differences in neural activity between conditions of interest is not as simple as controlling for effects of RT, or examining the overlap and separation of effects of RT and variables of interest. Instead, it is essential to have a theory that specifies whether and why differences between conditions should (or should not) be independent of RT in order to know how best to treat RT in neuroimaging studies. One framework that provides a way to relate cognitive processes to neural activity was set out by Taylor et al. (2013). We proposed that two principles govern the relationship between cognitive processes and aggregate measures of neural activity such as Blood Oxygenation Level Dependent (BOLD) fMRI: 1) engagement — stimuli that are represented by a model component or brain region should activate it more than stimuli that are not represented by a component or region; and 2) effort — within a set of stimuli that are represented by a model component or brain region; those that fit the representations less well should be more effortful to process, and thus produce greater activity, than those that fit the representations extremely well. As discussed in Taylor et al., the framework critically assumes that computational processes that are functionally separated in cognitive models can be mapped onto separate brain processes (Henson, 2005, Henson, 2006a, Henson, 2006b).

**Fig. 1:**
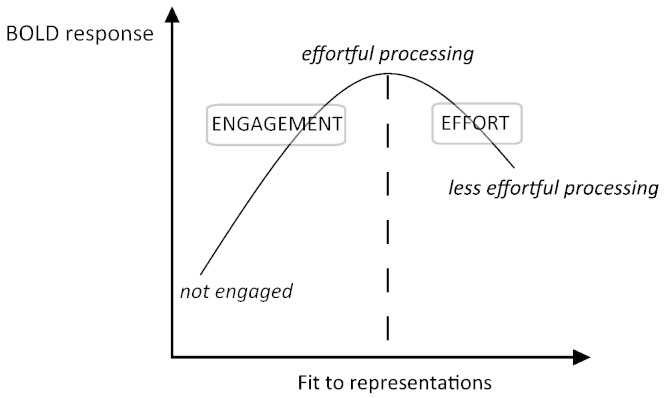
Inverted U-shaped function showing how engagement and processing effort relate to blood oxygenation level dependent (BOLD) signal.

As illustrated in Fig. 1, this proposal implies an inverted u-shaped relationship between the BOLD signal and the fit between stimuli and neural representations. The upward going portion of the curve is driven by greater engagement for stimuli which fit representations than for stimuli that do not. This is consistent with the majority of ‘subtraction’ studies in which differential neural activity is seen in regions that respond more to a preferred stimulus type than to other stimulus types. For example, a region in the right fusiform gyrus responds more strongly to faces than to other visual stimuli such as houses (Kanwisher et al., 1997), reflecting greater neural engagement for represented than non-represented stimuli. In contrast, the downward going portion of the inverted u-shaped function is driven by reduced effort for stimuli that fit the representations very well as compared to those that fit less well. This is consistent with repetition suppression or familiarity effects in functional imaging studies: highly familiar stimuli typically elicit reduced activity compared to less familiar stimuli (e.g., common versus uncommon orientations of an object), potentially due to sharpening of neural responses, or other mechanisms (Grill-Spector et al., 2006).

This inverted u-shaped relationship is thus needed to account for the existing functional imaging literature (see Taylor et al., 2013 for further details) and is related to other proposals of a non-linear relationship between the BOLD signal and cognitive processing, e.g., Price and Devlin (2011). A clear advantage of our proposal is that effort and engagement readily map onto cognitive distinctions (e.g., represented vs. non-represented stimuli, processing-time differences) that can be used to guide interpretation of neuroimaging contrasts, as detailed in the following paragraph.

**Fig. 2:**
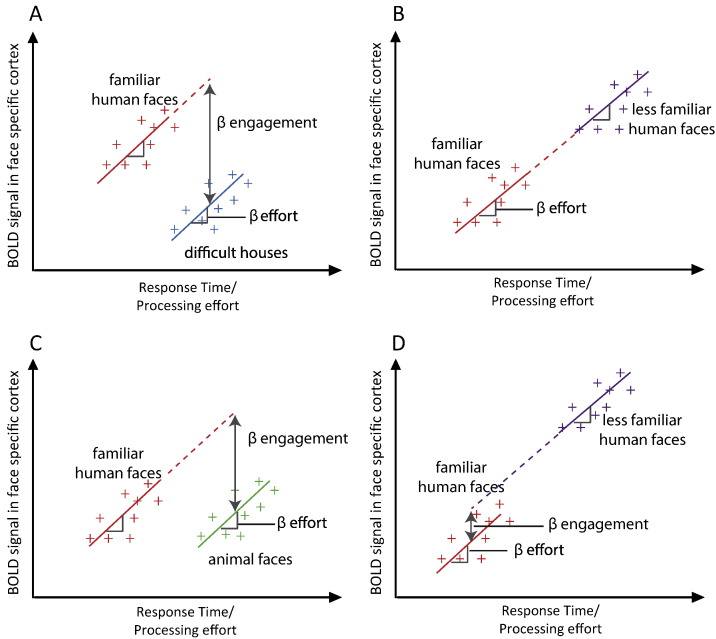
Plots showing four possible outcomes with regard to the relationship between RT and the BOLD response as measured by fMRI, in a hypothetical brain region specialized for processing faces. Graphs show response time and BOLD signal (scaling of a canonical HRF) for single trials and the interpretation of parameter estimates (β) from a general linear model fitted to this data. In all cases, two conditions differ in mean RT, and RT is correlated with BOLD signal. However, we can distinguish two sources of activation differences: β effort is the amount of change in BOLD signal per unit change in RT, calculated on the basis of all stimuli in the experiment. β engagement is the difference in BOLD signal between conditions, over and above the difference predicted from RT. We acknowledge that this approach presupposes that the relationship between RT and BOLD is the same in both conditions depicted. Inclusion of a condition-by-RT interaction term in statistical analysis allowed us to validate this assumption for the present data (reported in footnote ii), and would allow assessment of differential engagement even in the case of a significant interaction. In (A) and (C) β engagement is enhanced when response time differences are taken into account, in (B) and (D) β engagement is absent or reduced when response time differences are taken into account. These four profiles might be anticipated in the following circumstances: (A) faces engage/activate this brain region more than houses despite the fact that houses are more effortful/take longer to process. (B) Familiar and less familiar faces both engage this brain region but less familiar faces elicit greater activity entirely due to their longer RTs/greater processing effort. (C) Animal and human faces equivalently activate this brain region, but animal faces are more effortful to process, and accounting for these RT differences reveals the greater engagement of this region by faces. (D) Familiar and less familiar faces both engage this brain region, but less familiar faces elicit greater activity over and above that which would be expected on the basis of their longer RTs. This reveals that less familiar faces are both more effortful and engage an additional process relative to familiar faces.

Our framework suggests that a stimulus type that is represented by a particular brain region should engage that region more than another non-represented stimulus type. Here and throughout, we use the term “represents” to mean “represents some property of the stimulus”, for example, for written words this could be letters, phonemes, more basic visual or acoustic properties, as well as higher level conceptual information. These representations may be permanently instantiated in a neural system (e.g. specialized neurons that code for specific letters or words in posterior regions), or transient reading-related representations in frontal regions that serve related functions in other tasks (e.g. phonological output representations also used during object naming and spontaneous speech). This seems appropriate given that we are not committed to any particular representational system (e.g., localist versus distributed). As contrasts between represented and non-represented stimuli tap differences in engagement, clusters of activity revealed by such contrasts should survive correction for RT. However, if a brain region represents both stimulus types, then differential activity will be driven by processing effort and hence should positively correlate with RT. In such cases, correcting for RT should account for differential activity. Given these proposals, we can distinguish four possible outcomes in functional neuroimaging studies, as illustrated in Fig. 2, panels A to D.

(A) Greater activity is observed for a condition with faster/less effortful responses, indicating that this condition is associated with greater neural engagement. For example, if a particular cortical region contains face-specific representations, then faces will engage this region more than houses, even if the faces are very familiar and require minimal processing effort. In this case, effects of interest should be observed whether or not RT is entered as a covariate.
(B) Greater activity is observed for a condition with slower RTs, but entering RT as a covariate removes this between-condition difference in neural activity. This implies that the two conditions both engage the representations in a brain region, but one fits the representations less well. For example, familiar and less familiar faces should both engage face specific cortex but less familiar faces will have weaker representations and thus be more difficult for this region to process.
(C) A null effect is obtained when comparing two conditions unless RT is entered as a covariate. This outcome would occur if a region represents one stimulus type and not another, but the second engages that region to some extent (perhaps for some, but not all, stimuli, or only in certain situations) and is also more effortful to process. For example, face specific cortex may be somewhat engaged by animal faces and more engaged by human faces, but it may exert more effort in processing animal faces. In this case, entering RT as a covariate would enable the greater engagement by human faces to be observed.
(D) Greater activity is observed for a condition that is associated with slower or more effortful responses, even when RT effects are accounted for. For example, less familiar faces may be more effortful for face-specific cortex to process than familiar faces, but they may also engage an additional process, perhaps reflecting encoding of a new configuration of facial features. This outcome would provide evidence that differences in both effort and engagement contribute to activity in a region, and quantify their relative contributions. This outcome could only be observed by concurrently measuring behavioural and neural responses, and including an RT covariate in analyses.

Having laid out these general principles, in the next section, we use our framework to determine whether and why cognitive models of reading predict that contrasts between words and pseudowords, and irregular and regular words, should be independent of neural activity correlated with RT. Following this, we introduce a method for modelling contrasts of interest with and without RT as a covariate in a neuroimaging study of reading aloud, so as to derive region-specific estimates of neural engagement and effort. Finally, we describe an event-related fMRI study in which participants read aloud regular and irregular words and pseudowords whilst RT and accuracy were measured. Since comparisons between these stimuli are associated with RT differences (participants are slower to read irregular words and pseudowords than regular words), we can use these data both to illustrate a general method for co-varying RT differences in functional imaging analyses and to elucidate the role of engagement and processing effort in driving neural response differences during reading aloud.

### Engagement and effort during reading aloud

There are several computational models that explain how we read words, most prominently, the Dual Route Cascaded (DRC, Coltheart et al., 2001), triangle (Harm and Seidenberg, 2004, Plaut et al., 1996), and Connectionist Dual Process (CDP +, Perry et al., 2007) models. Whilst there are important theoretical and implementational differences between them, all three models propose that separable components represent item-specific knowledge, including representations of word meaning (all three models) and orthographic and phonological lexical representations (CDP + and DRC models), and generative knowledge of the relationship between spelling and sound. We can therefore derive largely overlapping predictions from these models as to whether particular brain regions should show differences in neural engagement or neural processing effort for words versus pseudowords and irregular versus regular words. More details about how we used the engagement and effort framework to derive these predictions are provided in Taylor et al. (2013) but they are outlined here briefly.

#### Words > pseudowords

In the DRC and CDP+ models, words have item-specific orthographic and phonological representations, as well as (as yet unimplemented) semantic representations, whereas pseudowords do not. In the triangle model, there are no item-specific orthographic or phonological representations, but semantic representations encode item-specific knowledge of word meanings, by which we mean that they allow words to be differentiated from each other and from similar looking pseudowords (see Harm and Seidenberg, 2004, Simulations 17–19). Words should therefore engage/activate brain regions that represent whole-word knowledge about orthographic and phonological form (DRC/CDP+ models) and/or semantics (all three models), more than pseudowords, which do not have item-specific representations. This should be the case despite the fact that pseudowords take longer to read. This can be understood by considering Fig. 2A, whereby words are akin to faces and pseudowords to houses — the former engage the relevant neural system to a greater extent than the latter, irrespective of any between-condition differences in RT. We should therefore obtain increased activity for words relative to pseudowords, reflecting engagement of brain regions representing item-specific information, whether or not we include RT as a covariate in our analyses.

#### Pseudowords > words

Although for somewhat different reasons (as outlined in Taylor et al., 2013), computational models of reading all suggest that the processes of mapping between spelling and sound and of resolving phonological output are more effortful for pseudowords than words. Therefore, activity should be greater for pseudowords than words in brain regions involved in performing such processes, but this should not be independent of RT. This prediction can be understood by considering Fig. 2B, whereby pseudowords are akin to less familiar faces and words to familiar faces. Both engage the relevant systems, but pseudowords are more effortful to process. We thus predict activity in areas involved in spelling–sound conversion and phonological output for pseudowords relative to words only in analyses which do not include RT as a covariate.

#### Irregular words > regular words

Computational models of reading aloud predict that brain regions involved in spelling–sound conversion and phonological output should be more active for irregular than regular words. Although the models differ in their precise explanations for this prediction, all three propose that it is due to increased processing effort; therefore, activation obtained for this contrast should correlate with RT. Again this can be related to Fig. 2B — irregular words correspond to less familiar faces and regular words to familiar faces. Thus, irregular > regular word activity should only be obtained in analyses that examine regularity effects without RT as a covariate.

#### Regular > irregular words

Regular words do not engage any computational model components more than irregular words, and they are less effortful for all components of the models to process. Therefore, we should not obtain activity for regular relative to irregular words in any brain regions relevant for reading aloud, irrespective of the treatment of RT (see Fig. 2B, regular words correspond to familiar faces, irregular words are less familiar faces).

### Modelling RT effects

In the current fMRI study, we examined activity during reading aloud of words versus pseudowords and irregular versus regular words. We explored the impact of accounting for RT variation, a proxy for differential processing effort, by constructing three different general linear models. First, we constructed a basic model that coded pseudowords, irregular words, regular words, and occasional errors as separate trials and did not include an RT regressor. Second, a lexicality model distinguished between neural effects of lexical status that were due to engagement and effort by including just two trial types, one for errors and one for correct responses, and adding two parametric modulators for the correct responses — one coding RT (in milliseconds — ms), and the other coding lexical status (1 for pseudowords or 0 for words). In this model, the RT (first) parametric modulator will capture neural response differences that are due to processing effort, whereas the lexical status (second) parametric modulator will capture differential engagement for words versus pseudowords, independent of RT differences. Third, we constructed a regularity model that included three trial types — errors, correct pseudoword responses, and correct word responses. Two parametric modulators were then included for word responses — one coding RT and the other regularity (1 for irregular words or 0 for regular words). In this model, processing effort for words will be captured by the RT parametric modulator, whereas the regularity parametric modulator will capture differential engagement for irregular vs. regular words, independent of RT. Thus, Model 1 allowed us to examine simple effects of lexical status and regularity, but did not separate neural response differences due to engagement and effort. In contrast, in Models 2 and 3, the default serial orthogonalisation procedures in the SPM software ensured that the RT (first) parametric modulator captured any shared between RT and lexical status (Model 2, second modulator) or regularity (Model 3, second modulator). Thus, examining the effect of the second parametric modulator in Models 2 and 3 allowed us to test for neural engagement differences due to lexical status and regularity respectively, over and above effects of processing effort (RT).

## Method

### Participants

22 (13 females) adults, who reported to be right-handed and native English speaking and were aged 18–40, took part in the experiment. Ethical approval was obtained from the Cambridge Psychology Research Ethics Committee, and informed consent was given by participants, who all reported to be neurologically healthy and did not have a history of reading or language impairments.

### Materials

60 regular words, 60 irregular words, and 60 pseudowords were selected from Rastle and Coltheart (1999). All items were monosyllabic, and the item sets were triplet-wise matched for the number of letters and initial phoneme (or phonetic class where exact phoneme was not possible) as this variable has a large effect on RTs in reading studies (Rastle et al., 2005). As the stimuli were selected from Rastle and Coltheart, the regular and irregular words were matched for Kucera and Francis (1967) frequency. However, using the SUBTLEX-UK Zipf frequency statistics (low frequency word values of 1 to 3, high frequency word values 4 to 7) (van Heuven et al., 2014), the irregular words (mean = 3.84, SD = .67) were higher in frequency than the regular words (mean = 3.38, SD = .54), t(174) = 4.95, p < .001.

### Imaging acquisition and analysis

Functional magnetic resonance imaging (MRI) data were acquired on a 3 T Siemens Trio scanner (Siemens Medical Systems, Erlangen, Germany) with a 12 channel head coil. Blood oxygenation level-dependent functional MRI images were acquired with fat saturation, 3 mm isotropic voxels and an interslice gap of .75 mm, flip angle of 78°, echo time [TE] = 30 ms, and a 64 × 64 data matrix. The acquisition was transverse oblique, angled to avoid the eyes and to achieve whole-brain coverage including the cerebellum. In a few cases the very top of the parietal lobe was not covered. We used a sparse imaging design with a repetition time (TR = 3500 ms) longer than the acquisition time (TA = 1940 ms), which provided a 1560 ms period in which to record spoken responses in the absence of echoplanar scanner noise (Edmister et al., 1999, Hall et al., 1999). This design also minimises the impact of head movement artefacts since images are not acquired whilst participants are speaking. Written words were presented in the centre of a white background, in black, 32 point Arial font, and were presented at the offset of the previous scan, i.e. at the beginning of the 1560 ms silent interval. Participants were instructed to read each word aloud as quickly and accurately as possible, and responses were recorded using a dual-channel MRI microphone, with noise cancelling software disabled since spoken responses were provided during silent periods between scans (FOMRI II, Optoacoustics). RTs were then coded offline by hand with the assistance of CheckFiles (a variant of CheckVocal, Protopapas, 2007), which enables wav files to be visualised and voice onsets to be marked and automatically recorded in a text file. In a single scanning run (12.6 min), 180 experimental trials were presented in a randomised order, split between ten 63 second blocks, each separated by three rest trials (10.5 s). To assist in anatomical normalisation we also acquired a T_1_-weighted structural volume using a magnetization prepared rapid acquisition gradient echo protocol (TR = 2250 ms, TE = 2.99 ms, flip angle = 9°, 1 mm slice thickness, 256 × 240 × 192 matrix, resolution = 1 mm isotropic).

Image processing and statistical analyses were performed using SPM8 software (Wellcome Trust Centre for Functional Neuroimaging, London, UK). The first 6 volumes of each scanning run were discarded to allow for equilibration effects. Images for each participant were realigned to the first image in the series (Friston et al., 1995). The transformation required to bring a participant's structural T1 image into standard Montreal Neurological Institute (MNI) space was calculated using tissue probability maps (Ashburner and Friston, 2005), and these warping parameters were then applied to all functional images for that participant. Normalised functional images were re-sampled to 2 mm isotropic voxels. The data were spatially smoothed with 8 mm full-width half maximum isotropic Gaussian kernel prior to model estimation.

Data from each participant were entered into three general linear models for event-related analysis (Josephs and Henson, 1999). In each model, events were convolved with the SPM8 canonical hemodynamic response function (HRF). Movement parameters estimated at the realignment stage of pre-processing were added as regressors of no interest. Low frequency signal drifts were removed with a high-pass filter (128 s) and AR1 correction for serial autocorrelation was made. In the first model there were 4 event types; regular word correct, irregular word correct, pseudoword correct, and errors. The second model was used to assess lexicality and RT effects and had two event types; errors and correct responses, and two parametric modulators on the correct response events; RT (in milliseconds), and pseudoword (1 or 0). The serial orthogonalisation employed by SPM when entering parametric modulators ensured that between-item variance due to slower pseudoword RTs was assigned to the RT parametric modulator. Therefore, only variance due to additional activity for pseudowords compared to words over and above these RT differences was fit using the pseudoword parametric modulator. Using a parametric modulator to account for RT variation is to assume that RT influences the magnitude, but not the timing or shape of the haemodynamic response. This is an appropriate assumption given the narrow range of response times in the present study (less than 100 ms between the fastest and slowest conditions). With a wider range of RTs a more complex model involving modulation of the first temporal derivative or dispersion might be appropriate. This could account for RT-related changes in the timing or duration of the haemodynamic response. The third model was used to assess regularity and RT effects and had 3 event types; errors, pseudowords, and words, and two parametric modulators on the word events; RT and irregular (1 or 0). As for the pseudowords in the second model, examining the effect of the second parametric modulator was equivalent to assessing additional activation for irregular compared to regular words when these items are matched for RT.

Contrasts of parameter estimates were taken forward to second level group analyses (one-sample and paired sample t-tests) using participants as a random effect. All comparisons were assessed using a voxel-wise uncorrected threshold of p < .001. After thresholding, only activations exceeding a cluster extent family wise error (FWE) corrected threshold of p < .05 were further considered for interpretation. Figures show results at this cluster extent corrected threshold, displayed on a canonical brain image. Since SPM employs zero-mean correction, such that the RT distribution for each participant is rescaled to have a mean of zero, graphs show signal change at specific voxels for an item with mean RT, with zero reflecting activity following un-modelled null events (in rest blocks). Cluster co-ordinates are reported in the space of the MNI152 average brain template and anatomical labels were generated by MRICron (Rorden et al., 2007) which uses the automated anatomical labelling (AAL) template (Tzourio-Mazoyer et al., 2002).

## Results

### Behavioural data

As shown in Table 1, accuracy was extremely high for all three stimulus types. RTs were faster for words than pseudowords, t_p_(21) = 6.25, p < .001, Cohen's d = 1.33, and for regular than irregular words, t_p_(21) = 4.03, p < .001, Cohen's d = .86.

**Table 1:** Accuracy and RT for all item types read aloud in the MRI scanner.

	Mean proportion of items correct (SD)	Mean RT for correct items in ms (SD)
Regular words	.998 (.007)	648 (83)
Irregular words	.980 (.021)	672 (93)
Pseudowords	.989 (.020)	721 (115)

### Neuroimaging data – Model 1 – without RT as a covariate

**Fig. 3:**
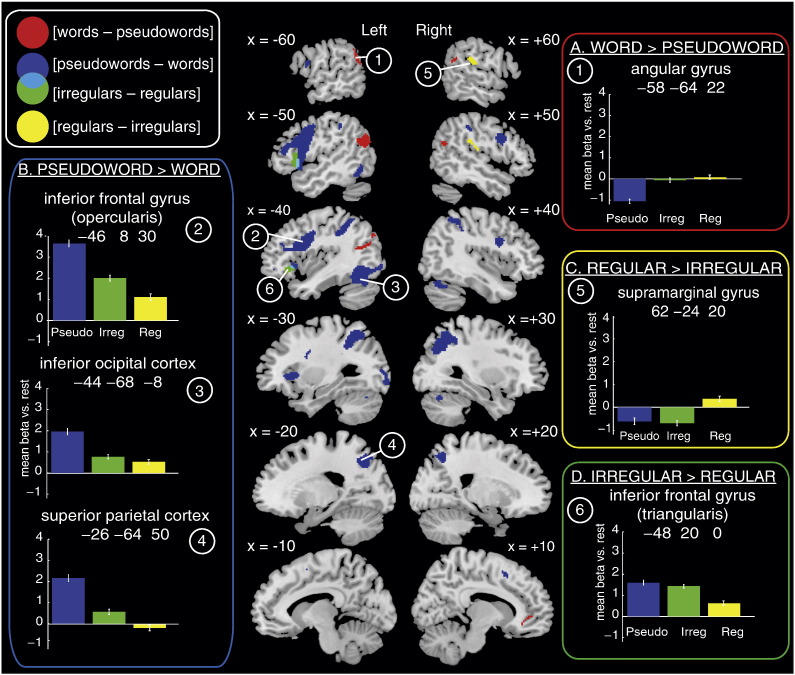
Brain regions showing differential activation for contrasts of interest in Model 1, which did not include an RT covariate. The left and right hemisphere slices show whole-brain activations at p < .001 voxel-wise uncorrected and p < .05 FWE cluster corrected for 22 participants. Red = [words − pseudowords], blue = [pseudowords − words], green = [irregular − regular words], yellow = [regular − irregular words], cyan = overlap between [pseudowords − words] and [irregular − regular words]. Panels contain plots showing activity (mean BOLD parameter estimate, arbitrary units) at peak voxels from contrasts of interests for each item type: blue = pseudowords, green = irregular words, yellow = regular words. All error bars in this and subsequent figures use standard error appropriate for within-participant designs (Loftus and Masson, 1994). Contrasts of interest represented by each panel are as follows: A) [words − pseudowords], B) [pseudowords − words], C) [irregular − regular words], D) [regular − irregular words].

**Fig. 4:**
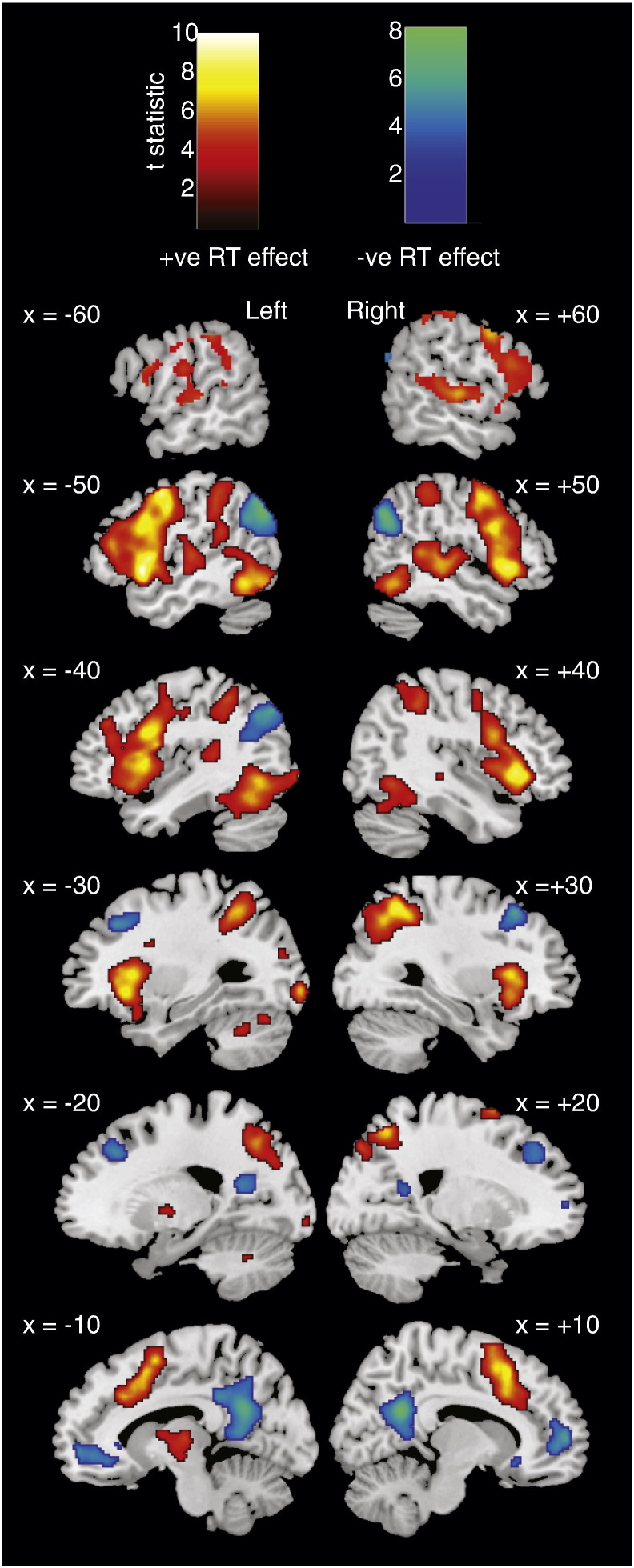
Brain regions showing a positive (hot colours) or negative (cool colours) correlation between BOLD signal and RT, collapsed across words and pseudowords, as derived from Model 2. Statistical maps were thresholded at p < .001 voxel-wise uncorrected and p < .05 FWE cluster corrected.

**Fig. 5:**
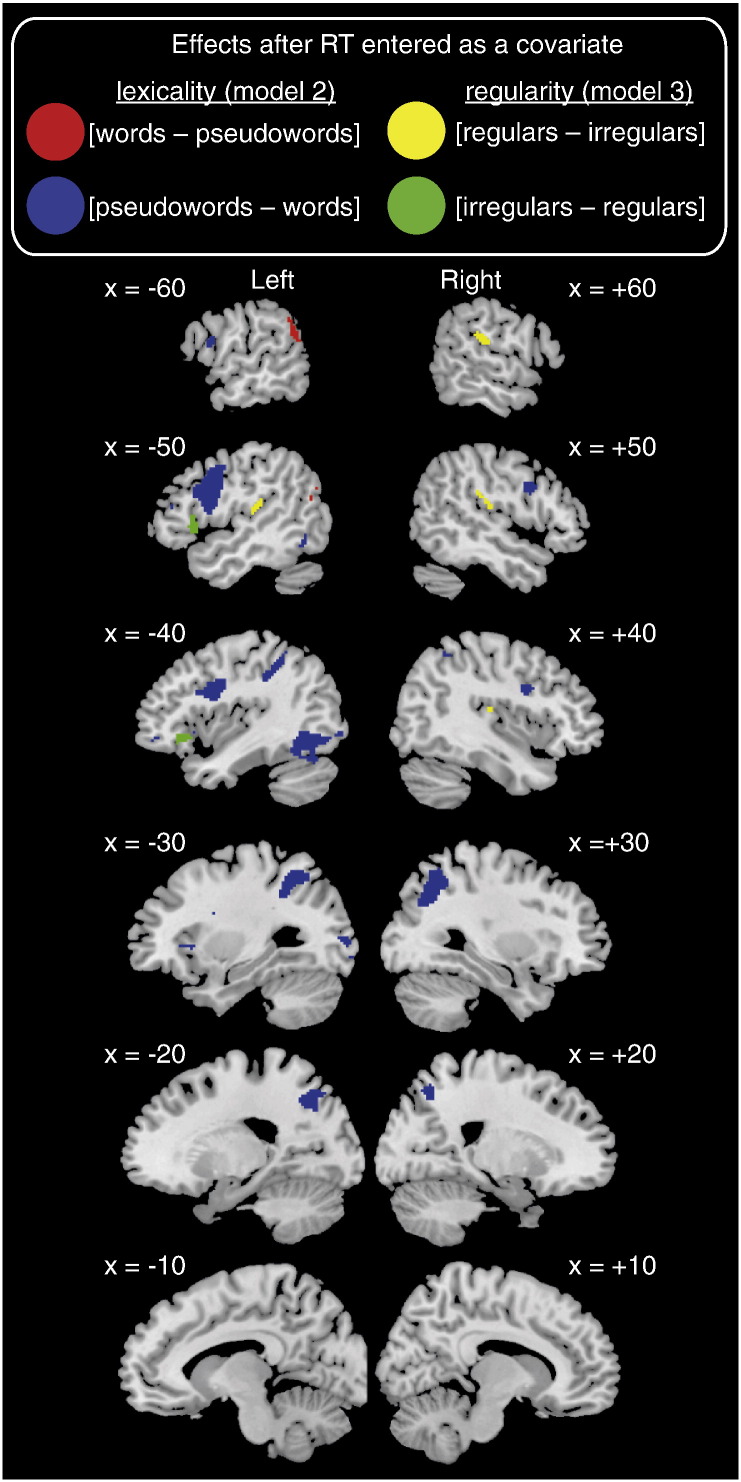
Brain regions showing differential activation for [words − pseudowords], [pseudowords − words] (both derived from Model 2), [irregular − regular words] and [regular − irregular words] (both derived from Model 3), over and above effects of RT. Colour coding and statistical thresholds as in Fig. 3.

Unless otherwise stated all reported results were significant at p < .001 whole brain voxel-wise uncorrected, and p < .05 cluster-level family wise error (FWE) corrected. Contrasts of parameter estimates are displayed on slices of the MNI canonical brain in Fig. 3, Fig. 4, Fig. 5.

#### Words > pseudowords (Table 2, Fig. 3A)

Activity was greater for word than pseudoword reading in bilateral middle temporal and angular gyri, anterior cingulate, and right supramarginal gyrus. However, inspection of response profiles shows that these regions were deactivated during reading relative to rest, with reduced deactivation for words than pseudowords.

**Table 2:** Brain regions activated in contrasts between words and pseudowords, and irregular and regular words in Model 1. p < .001 whole brain voxel-wise uncorrected, and p < .05 cluster-level FWE corrected. All peaks > 12 mm (lexicality) and 8 mm (regularity) apart are reported.

Region	Hemisphere	X	Y	Z	N voxels	Z	p
[Words − pseudowords]							
Middle temporal gyrus, angular gyrus, inferior parietal cortex, middle occipital cortex	L	− 58	− 64	22	856	4.31	< .001
		− 60	− 52	38			
		− 46	− 64	20			
		− 40	− 78	38			
Anterior cingulate, medial orbitofrontal cortex	Bilateral	− 8	26	− 8	827	4.13	< .001
		− 6	44	0			
		4	42	− 2			
		4	28	− 10			
		2	12	− 10			
		10	50	12			
Supramarginal gyrus	R	56	− 46	26	245	3.85	< .05
[Pseudowords − words]							
SMA	R	2	14	52	1202	5.89	< .001
Inferior frontal gyrus	R	48	8	20	645	5.86	< .001
		34	6	26			
		52	16	14			
Superior and inferior parietal cortex, middle occipital cortex, supramarginal gyrus	R	32	− 58	50	1512	5.6	< .001
		30	− 70	32			
		50	− 28	40			
		34	− 40	40			
		40	− 42	54			
Inferior frontal gyrus, insula, precentral gyrus	L	− 46	8	30	3561	5.59	< .001
		− 32	20	0			
		− 54	8	16			
		− 48	36	18			
		− 36	28	− 10			
		− 54	16	− 4			
		− 46	50	− 8			
		− 34	26	20			
Inferior and superior parietal cortex, superior occipital cortex	L	− 26	− 64	50	1737	5.35	< .001
		− 36	− 52	54			
		− 46	− 38	44			
		− 24	− 72	34			
Cerebellum, inferior and middle occipital cortex	L	− 46	− 58	− 24	1572	4.52	< .001
		− 44	− 68	− 8			
		− 38	− 86	− 6			
		− 28	− 96	− 4			
		− 32	− 90	6			
		− 30	− 68	− 26			
Cerebellum	R	28	− 66	− 26	380	4.5	< .01
		46	− 62	− 24			
[Irregular − regular words]							
Inferior frontal gyrus	L	− 48	20	0	518	4.2	< .001
		− 40	30	− 6			
		− 52	18	12			
		− 40	34	− 14			
[Regular − irregular words]							
Heschl's gyrus, supramarginal gyrus	R	44	− 20	14	421	6.01	< .01
		62	− 24	20			
		54	− 22	20			
		54	− 30	26			

#### Pseudowords > words (Table 2, Fig. 3B)

Activation was greater for pseudowords than words in the supplementary motor cortex, bilateral inferior frontal and precentral gyri (more extensive on the left), left insula, bilateral superior and inferior parietal cortices, left inferior occipital and inferior temporal cortices, and bilateral cerebellum. In all regions, activity was greater for reading relative to rest.

#### Regular words > irregular words (Table 2, Fig. 3C)

Regular words activated right supramarginal and Heschl's gyri more than irregular words.

#### Irregular words > regular words (Table 2, Fig. 3D)

This contrast revealed activity in the left inferior frontal gyrus (IFG — triangularis and orbitalis), which somewhat overlapped with activity greater for pseudowords than words.

### Neuroimaging data – Model 2 – Lexicality effects with an RT covariate

#### Effects of RT (Table 3, Fig. 4)

To reveal brain activity correlated with RT for all item types combined, we examined the effect of the first parametric modulator in this second model. Positive correlations with RT were obtained in a large network of regions, including bilateral occipitotemporal and parietal cortices, bilateral inferior frontal and precentral gyri, insula and superior temporal pole, supplementary motor cortex, and bilateral middle temporal gyri. Negative correlations between brain activity and RT were obtained in anterior cingulate, precuneus, and bilateral angular gyri.^4^

**Table 3:** Brain regions where activity correlated with RT in Model 2. p < .001 whole brain voxel-wise uncorrected, and p < .05 cluster-level FWE corrected. All peaks > 12 mm apart are reported.

Region	Hemisphere	X	Y	Z	N voxels	Z	p
*Positive RT correlations*							
Precentral gyrus, superior temporal pole, inferior frontal gyrus, inferior temporal gyrus, insula, parietal cortex, superior and inferior occipital cortex, postcentral gyrus, cerebellum	L	− 54	− 2	44	11,862	6.02	< .001
		− 52	12	− 4			
		− 42	10	26			
		− 48	− 54	− 14			
		− 42	12	4			
		− 28	22	6			
		− 28	24	− 6			
		− 32	− 50	48			
		− 26	− 94	− 4			
		− 46	− 66	− 12			
		− 54	32	12			
		− 22	− 64	48			
		− 46	− 40	46			
		− 54	24	26			
		− 58	− 32	44			
		− 22	− 74	36			
		− 42	32	22			
		− 56	− 16	− 2			
		− 54	− 16	20			
		− 26	− 56	− 32			
SMA, medial superior frontal gyrus, cingulate	Midline	− 6	10	50	3400	5.95	< .001
		6	18	48			
		− 6	20	40			
		16	10	68			
		2	30	46			
		6	26	28			
Insula, putamen, precentral gyrus, inferior frontal gyrus, superior and middle temporal gyrus, post- and pre-central gyrus	R	46	22	− 2	6322	5.81	< .001
		34	22	4			
		52	2	44			
		48	10	24			
		52	− 24	2			
		54	− 40	8			
		50	12	12			
		64	− 4	24			
		58	− 8	0			
		36	− 2	52			
		70	− 26	8			
Angular gyrus, superior and inferior parietal cortex, supramarginal gyrus, cuneus	R	30	− 56	50	2098	5.56	< .001
		20	− 62	54			
		54	− 36	48			
		38	− 42	44			
		58	− 24	50			
		20	− 80	44			
Inferior temporal gyrus, cerebellum, fusiform gyrus	R	48	− 58	− 12	842	4.92	< .001
		38	− 66	− 24			
		44	− 56	− 32			
		38	− 44	− 20			
Thalamus	L	− 8	− 16	4	369	4.15	< .01
		− 14	− 4	6			

*Negative RT correlations*							
Precuneus and cingulate	Midline	8	− 54	24	2952	5.49	< .001
		− 2	− 58	40			
		− 4	− 38	38			
		12	− 40	26			
Angular gyrus, and middle occipital cortex	L	− 50	− 68	34	1063	5.23	< .001
		− 40	− 74	40			
		− 38	− 56	22			
Angular gyrus	R	50	− 64	32	558	5.2	0.001
Middle frontal gyrus	L	− 24	32	40	426	5.05	< .01
		− 26	16	42			
Middle and superior frontal gyrus	R	28	22	46	442	4.82	< .01
		22	38	42			
Medial orbitofrontal cortex,	Midline	0	38	− 12	1958	4.73	< .001
Medial superior frontal		6	56	6			
Gyrus, anterior cingulate,		2	56	− 8			
White matter		− 4	26	− 8			
		− 8	46	− 6			
		− 6	28	4			
		0	4	− 8			
		− 4	− 16	8			

#### Effects of lexical status (Table 4, Fig. 5)

To determine whether there were differences in activity between words and pseudowords that were independent of RT, we examined the effect of the second parametric modulator (pseudoword: 1 or 0). Fig. 5 (blue) shows that, after the effects of RT were accounted for, pseudowords relative to words activated bilateral (although more extensive on the left) inferior frontal and precentral gyri, bilateral superior and inferior parietal cortices, supplementary motor cortex, and left inferior occipital cortex, posterior fusiform and cerebellum. Words relative to pseudowords activated both left angular and middle temporal gyri over and above RT effects (Fig. 5, red).^5^

**Table 4:** Brain regions showing a significant lexicality effect (second parametric modulator) over and above the RT effect (first parametric modulator) in Model 2. p < .001 whole brain voxel-wise uncorrected, and p < .05 cluster-level FWE corrected. All peaks > 12 mm apart are reported.

Region	Hemisphere	X	Y	Z	N voxels	Z	p
[Words − pseudowords]							
Angular gyrus, supramarginal gyrus, middle temporal gyrus, middle occipital cortex	L	− 62	− 54	24	357	4.49	< .01
		− 60	− 50	40			
		− 56	− 66	20			
		− 42	− 70	24			
[Pseudowords − words]							
Precentral gyrus, inferior frontal gyrus	L	− 46	6	32	1569	5.16	< .001
		− 58	10	16			
		− 48	36	16			
Superior parietal cortex	R	30	− 58	50	917	5.15	< .001
		32	− 70	34			
Superior and inferior parietal cortex	L	− 26	− 64	50	1372	5.09	< .001
		− 36	− 52	54			
		− 30	− 48	42			
		− 40	− 42	48			
Superior occipital cortex	L	− 24	− 72	34			
		− 28	− 68	16			
Precentral and inferior frontal gyrus	R	48	8	30	352	5.07	< .01
		36	6	28			
SMA	R	2	14	52	596	4.85	< .001
Inferior occipital cortex, cerebellum, middle and inferior occipital cortex, fusiform gyrus	L	− 44	− 68	− 8	1064	4.3	< .001
		− 46	− 62	− 22			
		− 34	− 88	6			
		− 40	− 52	− 14			
		− 38	− 86	− 6			
		− 26	− 96	− 6			
Insula inferior frontal gyrus	L	− 34	20	0	263	4.24	< .05
		− 38	38	− 6			
		− 44	50	− 8			

### Neuroimaging data – Model 3 – Regularity effects with an RT covariate

#### Effects of RT

In Model 3, the RT parametric modulator was only applied to the regressor coding correct responses to words, whereas the RT effects obtained from Model 2 were for words and pseudowords combined. However, as expected, the RT effects were very similar for all items and for words alone. A table of peak co-ordinates for RT effects in Model 3 is provided in the Supplementary materials.

#### Effects of regularity (Table 5, Fig. 5)

Fig. 5 (green) shows that we observed activity for irregular relative to regular words (second parametric modulator) after RT effects (first parametric modulator) were accounted for in anterior portions of the left IFG (orbitalis and triangularis). The reverse contrast (yellow) revealed that bilateral rolandic operculum, right supramarginal gyrus, and left superior temporal gyrus were more active for regular than irregular words once RT effects had been accounted for.

**Table 5:** Brain regions showing a significant regularity effect (second parametric modulator) over and above the RT effect (first parametric modulator) in Model 3. p < .001 whole brain voxel-wise uncorrected, p < .05 cluster-level FWE corrected. All peaks > 8 mm apart reported.

Region	Hemisphere	X	Y	Z	N voxels	Z	p
[Irregular–regular words]							
Inferior frontal gyrus	L	− 48	20	0	334	4.09	< .01
		− 40	26	− 6			
		− 50	20	10			
		− 56	16	16			
		− 40	34	− 14			
[Regular–irregular words]							
Rolandic operculum, supramarginal gyrus	R	44	− 20	16	471	4.72	0.001
		62	− 24	20			
		54	− 22	20			
		54	− 30	26			
Rolandic operculum superior temporal gyrus	L	− 48	− 22	14	224	3.88	< .05
		− 54	− 28	18			
		− 48	− 16	20			

### Analysis of prefrontal cortex regions of interest (Fig. 6)

**Fig. 6:**
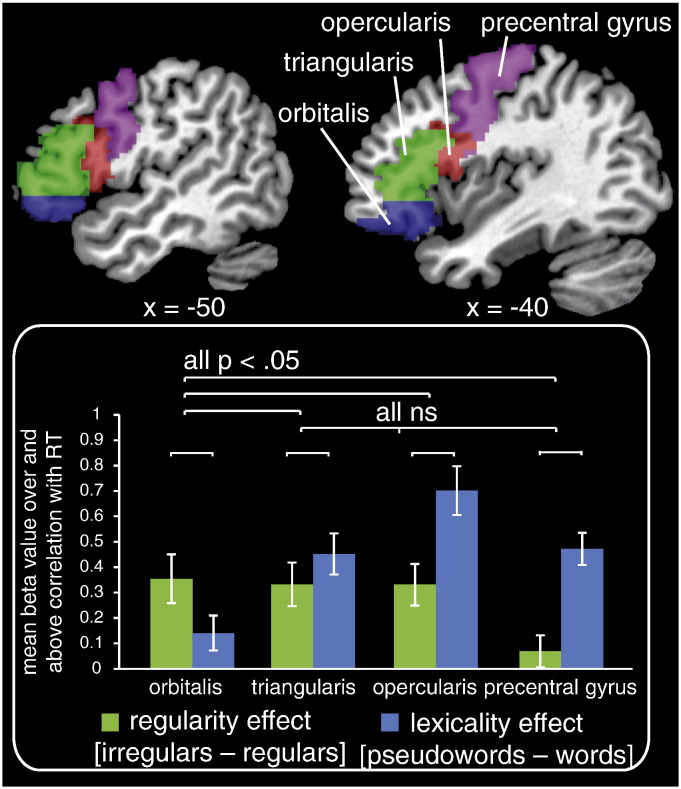
The two left hemisphere slices showing four prefrontal cortex regions of interest (ROIs) defined using the AAL template. Plot shows the mean beta value in each of these ROIs for [pseudowords − words] (blue bars — Model 2 second parametric modulator) and [irregular − regular words] (green bars — Model 3 second parametric modulator), over and above activation correlated with RT (first parametric modulator in each model). Brackets show the comparisons that were tested in post-hoc 2 (region) × 2 (contrast) ANOVAs.

In analyses that contrasted item types without considering RT differences (Model 1, Fig. 3) we observed overlapping prefrontal cortex activation for pseudowords relative to words and irregular relative to regular words. In line with our meta-analysis (Taylor et al., 2013), this suggests that common regions of the left IFG are activated by these two contrasts that tap phonological output effort. However, Fig. 5 shows that when we exclude activation that is associated with RT, some separation is obtained between activation for pseudowords relative to words (Model 2) and irregular relative to regular words (Model 3). To confirm this differentiation, we conducted a repeated measures ANOVA to contrast the effects of lexical status and regularity over and above RT (the second parametric modulators from Models 2 and 3 respectively) in four anatomically defined regions of interest (ROIs) in prefrontal cortex, which were obtained from the AAL template (Tzourio-Mazoyer et al., 2002), as depicted in Fig. 6. This contrast (lexical status vs. regularity) by region (IFG — orbitalis vs. triangularis vs. opercularis vs. precentral gyrus) ANOVA obtained a main effect of region, F(3, 63) = 7.47, p = .001, whereby activation for both contrasts was greater in IFG triangularis than all other regions, and no main effect of contrast, F(1,21) = 2.01, p = .17. Importantly, it also revealed a contrast by region interaction, F(3,63) = 11.40, p < .001. To determine the source of this interaction effect, we conducted six two-by-two post-hoc ANOVAs to determine which of the four regions differed from each other with respect to the regularity vs. lexicality contrast. When Bonferroni corrected for multiple comparisons, an interaction between region and contrast was obtained in the post-hoc ANOVAs that compared IFG orbitalis with each of the other three regions, but not in the ANOVAs that compared these three regions with each other (Table 6 shows the results of these post-hoc ANOVAs). As is clear from the plot in Fig. 6, the regularity effect is numerically larger than the lexicality effect in the left IFG orbitalis, but the reverse is true in the other three regions.

**Table 6:** Post-hoc region × contrast ANOVAs comparing each of the four regions with each other, with respect to the lexicality versus the regularity effect.

Region	Contrast × region interaction		
	F statistic	Corrected p value	Effect size
Orbitalis vs. triangularis	8.57	< .05	0.29
Orbitalis vs. opercularis	18.24	< .01	0.47
Orbitalis vs. precentral gyrus	27.87	< .001	0.57
Triangularis vs. opercularis	8.2	.06	0.28
Triangularis vs. precentral gyrus	4.52	ns	0.18
Opercularis vs. precentral gyrus	< 1	ns	< .1

## Discussion

In cognitive neuroscience research, concerns have been raised that contrasts between conditions of interest are often confounded by associated RT differences. However, the engagement and effort framework (Taylor et al., 2013) provides criteria for predicting whether and how RT variation should be associated with neural activity. Between-condition differences in neural activity *should only be independent of RT* if it is hypothesised that a brain region represents one stimulus type but not another. In this case differential activity should reflect greater engagement of this region by the preferred stimulus type. As noted in the introduction, the term “represents” is here taken to mean, “represents some property of the stimulus in the context of the present task”. In contrast, RT *should correlate* with between-condition differences in neural activity if two stimulus types are both represented by a brain region, but one fits the representations in that region better than the other and therefore requires less processing effort.

To illustrate the value of this framework, we tested predictions from cognitive models as to whether differences in activity during word versus pseudoword and irregular versus regular word reading should be independent of RT. We now discuss the results of these analyses, using the four potential functional imaging outcomes predicted by the engagement and effort framework (as depicted in Fig. 2) to structure this discussion.

### Activity is greater for a condition associated with shorter RTs (Fig. 2A)

**Fig. 7:**
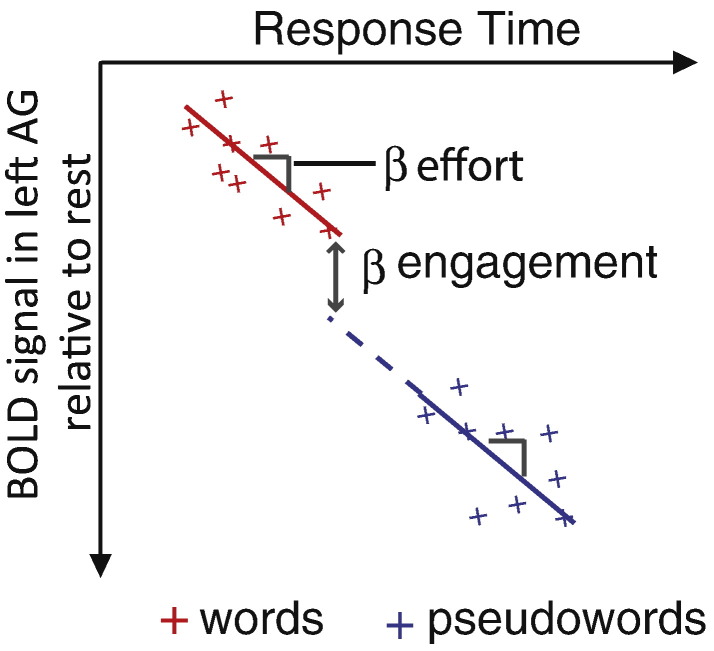
Plot illustrating the nature of response profile (but not actual data) in the left angular gyrus for a set of hypothetical words and pseudowords that vary in RT. β effort is the amount of change in BOLD signal per unit change in RT (across all items), i.e. more deactivation for longer RTs. β engagement reflects the residual BOLD signal we observed for words over pseudowords, after accounting for the negative correlation between activity and RT, i.e. more engagement for words than pseudowords.

Our framework specifies that stimuli that are represented by a particular brain region should engage/activate that region more than stimuli that are not represented, independent of any between-condition differences in RT. Cognitive models of reading predict that we should obtain this response profile for the contrast [words − pseudowords] in brain regions representing semantic (triangle, DRC, and CDP+ models) or whole-word orthographic or phonological information (DRC and CDP+ models only), because words have such representations whereas pseudowords do not (see Taylor et al., 2013 for further details). Support for this prediction was provided by the finding that the contrast [words − pseudowords] revealed activation in the left angular gyrus (AG), both before (Model 1) and after (Model 2) RT was entered as a covariate, despite the fact that words were read faster than pseudowords. However, activity in the left AG was not in fact independent of RT, as in the situation depicted in Fig. 2A, but negatively correlated with it and this region was deactive during reading aloud relative to rest. Nonetheless, left AG deactivation for words relative to pseudowords was less than would have been expected on the basis of the shorter RTs obtained for words. This region's activity profile is illustrated by the plot shown in Fig. 7.

The left AG is part of the “default mode” network that often deactivates during tasks relative to rest (Buckner et al., 2008, Gusnard and Raichle, 2001). Binder (2012) proposed that part of this resting state activity reflects semantic processing, because regions typically active during the resting state (Binder et al., 1999), including the left AG, were also highlighted in a meta-analysis of semantic processing (Binder et al., 2009). Applying this perspective to our data, we suggest that word reading deactivated the left AG less than pseudoword reading, over and above RT differences between the two conditions, because words are meaningful and engage some of the semantic processes that this region carries out during the resting state. However, it should be noted that the nature of processing during the resting state is not well understood and that alternative explanations have been proposed for left AG function (Seghier, 2013).

### Between-condition differences in activity are not independent of associated RT differences (Fig. 2B)

The only regions in which between-condition differences in activity were entirely correlated with RT (i.e., activity was only observed in Model 1 which did not have an RT covariate) were the right angular gyrus and anterior cingulate cortex, for the contrast [words − pseudowords]. These regions showed a negative correlation between activity and RT and were more deactive during pseudoword than word reading. As these regions form part of the default mode network, deactivation during reading aloud was likely entirely driven by processing effort, which detracted from task-irrelevant processes that these regions are engaged in when at rest, e.g., self-reflective thought, emotional processing, remembering the past and envisioning the future (Buckner et al., 2008). Although the left AG is also part of this network and likely engaged in similar processes, ROI analyses indicated that the response profile in this region differed significantly from that in the right AG. Using left and right AG ROIs defined using the AAL template, [word − pseudoword] activity over and above RT was significantly greater in the left than in the right AG, t(21) = 3.18, p < .01, and was significantly different from zero in the left, t(21) = 1.82, p < .05, but not in the right AG, t(21) < 1, ns. We therefore suggest that a greater proportion of resting state activity reflects semantic processing in the left than in the right AG, and hence words *engage* the left, but not the right AG more than pseudowords.

### Between-condition differences in activity are masked by associated RT differences (Fig. 2C)

If a brain region represents one stimulus type and not another, but the second stimulus type engages that brain region to some extent *and* is more effortful to process, this greater processing effort could mask greater engagement by the first stimulus type. However, this greater engagement should be revealed when RT is taken into account, as depicted in Fig. 2C. The only regions in which we obtained this response profile were the left rolandic operculum and superior temporal gyrus, in which cluster corrected activity was observed for the contrast [regular − irregular words] in Model 3 (after RT was entered as a covariate) but not in Model 1 (without RT entered as a covariate). These regions may have been activated due to participants processing the sound of their own voice when reading aloud, as shown previously during picture naming or propositional speech (Christoffels et al., 2007, Dhanjal et al., 2008), consistent with models of speech production that include auditory feedback control systems (Guenther et al., 2006, Price et al., 2011). This is also likely to be the case for their right hemisphere homologues in which we obtained regular relative to irregular word activity both before (Model 1) and after (Model 3) RT was taken into account. Such processes are beyond the scope of cognitive models of reading, and it is not entirely clear why regular words should engage such mechanisms more than irregular words. We therefore reserve judgement as to what drove the profile of activity we observed until the current results can be replicated in subsequent studies.

### Between-condition differences in activity are obtained over and above associated RT differences (Fig. 2D)

Unlike some previous neuroimaging studies of reading (Binder et al., 2005, Graves et al., 2010), our framework does not suggest that differences in task difficulty (as indexed by RT) between pseudowords and words, and irregular and regular words, should largely be reflected by activation differences in brain regions involved in domain-general cognitive processes. Instead, cognitive models of reading aloud predict that such contrasts tap increased processing effort in regions involved in spelling-to-sound conversion and phonological output, and thus activity revealed by these contrasts *should* be entirely correlated with RT. Consistent with this, we obtained strong positive correlations between activity and RT in the left inferior/superior parietal cortex, a region ostensibly involved in spelling-sound conversion, and the left inferior frontal gyrus, a region involved in phonological output, as well as in the left occipitotemporal cortex, a region involved in orthographic processing (see Taylor et al., 2013 for evidence supporting these region-to-function attributions).

Unexpectedly, in the left occipitotemporal cortex, left inferior/superior parietal cortex, and left inferior frontal gyrus we obtained pseudoword relative to word activity over and above effects of RT, and the left inferior frontal gyrus was also more active for irregular relative to regular words, over and above RT. According to the effort and engagement framework, residual activity for pseudowords and irregular words relative to regular words (over and above positive correlations with RT) indicates that these item types were not only more effortful for certain brain regions to process, but also that they engaged the representations in these regions to a greater extent (as illustrated in Fig. 2D). It is only by entering RT as a covariate in our analyses that we could have discovered this. In the following paragraphs we outline what might account for the greater engagement of these regions by certain item types. However, we must first acknowledge an alternative explanation for the residual activity observed, which is that our RT measure was not a precise proxy for processing effort. Specifically, this implies that RT systematically under-estimated processing effort for pseudowords relative to words and irregular relative to regular words, rather than that RT systematically overestimated processing effort, or that RT measurements were generally noisy. Against this suggestion, we derived neural predictions from cognitive models of reading aloud for which RT is the key dependent variable, and thus our RT measure should at least have captured processing effort as envisaged in these models. At present we know of no better way of estimating processing effort than by using RT, since RT is a direct consequence of the time-course of the processes performed in order to achieve a response, unlike other measures such as galvanic skin response or pupil dilation. However, further examination of the relationship between RT and processing effort is required. With this caveat in mind, we now discuss possible functional explanations for the activation clusters we obtained that survived correction for RT.

RT correlated pseudoword relative to word activity in the left occipitotemporal cortex may have been driven by the greater effort of processing less orthographically typical forms, as words had a significantly higher neighbourhood size (Coltheart N = 5.57, SD = 4.94) than pseudowords (Coltheart N = 4.11, SD = 3.94), t(262) = 2.42, p < .05. However, Taylor et al.'s (2013) meta-analysis confirmed pseudoword > word activation in this region when only studies with relatively well matched words and pseudowords were included. Furthermore, two studies have failed to obtain neighbourhood size effects in the left OT (Binder et al., 2003, Fiebach et al., 2007). However, Vinckier et al. (2007) did find this region to be sensitive to orthographic typicality, as measured by bigram and quadrigram frequency, thus it remains possible that orthographic differences between words and pseudowords were in part responsible for the pseudoword > word RT correlated effects in the left OT.

Left OT activation that was greater for pseudowords than words over and above these RT correlated effects may reflect top-down signals from regions that translate letters to sounds (left parietal cortex) and compute phonological output (left inferior frontal gyrus) (Price and Devlin, 2011, Taylor et al., 2013). Such feedback may be generated on-line during the process of reading aloud or may reflect post-response learning processes that help encode the orthographic form of pseudowords for longer retention. This idea is embodied in Share's (1995) self-teaching hypothesis, which suggests that the process of effortful decoding (i.e., mapping from spelling to sound), facilitates the acquisition of word-specific orthographic information. Such an orthographic learning mechanism has also recently been implemented in a computational model, which builds an orthographic lexicon through repeated attempts to read words aloud (Ziegler et al., 2014).

Greater activation of the left parietal cortex by pseudowords than words, over and above positive correlations between activity and RT, may reflect engagement of pre- and/or post-response mechanisms that learn about pseudoword letter-sound correspondences in order to improve performance if the item is encountered in the future. This is supported by findings showing that such learning processes occur when adults listen to and repeat novel spoken words (Davis et al., 2009, Gaskell and Dumay, 2003), and that activity in the left inferior parietal cortex changes when people learn new visual–verbal associations (Breitenstein et al., 2005, Hashimoto and Sakai, 2004) or new artificial orthographies (Taylor et al., in press).

Finally, ROI analyses indicated that, over and above activity positively correlated with RT, pseudowords relative to words more strongly activated posterior and dorsal aspects of the left IFG and precentral gyrus, whereas irregular relative to regular words more strongly activated ventral and anterior aspects of the left IFG. This suggests that, when phonological output effort is excluded by our method of modelling RT-correlated activity, irregular and pseudoword reading, relative to regular word reading, engage separable regions in prefrontal cortex.

Existing research particularly implicates posterior regions of the left IFG and precentral gyrus in phonological/articulatory processing (Bookheimer, 2002, Devlin et al., 2003, Gough et al., 2005, Poldrack et al., 1999) and phonological memory (Mechelli et al., 2007, Owen et al., 2005), and neuropsychological data indicate that damage to this region can have negative consequences for pseudoword reading (Fiez et al., 2006, Woollams and Patterson, 2012). Davis et al. (2009) found that activity in the left precentral gyrus changed as adults were familiarised with and consolidated the phonological forms of novel words. It may therefore be that these regions were engaged by pseudowords more than words because pre- and/or post-response learning took place about the novel phonological forms being pronounced.

With regard to greater engagement of the left anterior IFG for irregular relative to regular words, some have argued that this reflects the recruitment of semantic representations, in line with the idea that this region is involved in controlled semantic retrieval (Badre and Wagner, 2002, Wagner et al., 2001). For example, Graves et al. (2010, p1809) obtained activity in this region that was negatively correlated with spelling–sound consistency during reading aloud, and suggested that this reflected “top-down attentional modulation of semantic networks in the MTG/ITS”. This perspective is motivated by the triangle model (Harm and Seidenberg, 2004, Plaut et al., 1996), in which “lesions” to semantic representations are more detrimental for irregular than regular word reading. However, as elaborated on by Taylor et al. (2013, p 9, p19), the primary goal of reading is to access meaning and, thus, connections from orthography to semantics should be equivalently strong for all words (Harm and Seidenberg, 2004, Simulation 11). Thus, whilst semantic representations may be necessary for irregular but not regular word naming, engagement/activation of these representations should not be greater for irregular than regular words in typical adult readers. Overall, although we cannot rule out the idea that irregular relative to regular word activity, over and above RT effects, in the left IFG orbitalis reflects engagement of semantic representations, we do not believe that this perspective is motivated by existing cognitive theories of reading.

It has been proposed that progressively anterior regions of prefrontal cortex support increasingly complex representations and processes (Badre, 2008). For example, Koechlin and Summerfield (2007) argued that posterior lateral prefrontal cortex (including IFG orbitalis and triangularis) is involved in selecting actions that are not only driven by the immediate sensory input, but require taking context into account. Applying this perspective to our data, given only the orthography of an irregular word, multiple possible pronunciations are possible: the incorrect regularised pronunciation that can be derived directly from the letters in the word, and the correct pronunciation that must be retrieved from memory. Thus, irregular word reading may engage multiple phonological representations, an idea supported by behavioural priming investigations (Ranbom and Connine, 2011, Taft et al., 2008).

Nosarti et al. (2010) suggested a similar explanation for their finding that adults' second language proficiency positively correlated with activation in the left IFG orbitalis during first language reading. Specifically they proposed that learning a second language increases conflict between spelling and sound because letters are pronounced differently in the two languages. The idea that irregular word reading requires selection between multiple phonological representations has also been put forward in the developmental literature. Tunmer and Chapman (2012) found that children's ability to understand and correct mispronunciations of spoken words (e.g., BUSH pronounced to rhyme with RUSH), indicative of flexible lexical phonological representations, predicted their irregular word reading accuracy, after controlling for phonemic awareness, vocabulary knowledge, and pseudoword reading skill. Overall this research suggests that irregular words may activate anterior portions of the left IFG more than regular words because they engage multiple phonological representations that must be selected between.

## Summary and conclusions

### Advancements to our understanding of the neural systems for skilled reading

By using the engagement and effort framework and conducting contrasts between words and pseudowords and irregular and regular words both with and without RT as a covariate, we validated the dual-pathway left occipitotemporal and parietal system for reading aloud, as shown in Figure 8 of Taylor et al. (2013). However, we also obtained several novel findings that should advance our understanding of the neural architecture of skilled reading, as summarised in the following paragraphs.

Results from our word − pseudoword contrast clearly demonstrated the value of conducting analyses with and without RT as a covariate. Unlike the right angular gyrus and anterior cingulate, which deactivated during reading relative to rest in a manner that entirely covaried with RT, the left angular gyrus was *less* deactivated by words relative to pseudowords than would be predicted from this region's negative activity-RT correlation. If the left angular gyrus is engaged in semantic processing when at rest (Binder et al., 2009; Binder, 2012), our data specifically implicate this region in processing/retrieving word meanings during reading aloud.

Our results further showed that pseudowords relative to words activated left posterior inferior frontal, parietal, and occipitotemporal cortices, over and above positive correlations between activity and RT. This suggests that pseudowords are not only more effortful in terms of orthographic processing, spelling–sound conversion, and phonological output, in line with cognitive models of reading, but also engage some representation or process in these regions more than words. At present we speculate that pseudoword reading may engage learning mechanisms that encode information about novel phonological and orthographic forms and the mappings between them. Future research should therefore investigate whether learning does indeed take place each time a pseudoword is read, drawing on methods employed in the spoken word learning literature (Breitenstein et al., 2005, Davis et al., 2009). Such studies could also use the temporal resolution provided by magnetoencephalography and electroencephalography (MEG/EEG) to determine whether such learning processes occur pre- or post-naming. This research could be complemented by extending current computational models, such as the DRC, triangle, and CDP + models of reading, to incorporate on-line learning mechanisms, and investigate the impact of recent experience with reading pseudowords on activity in all components of these models.

Our final novel finding was that irregular relative to regular word reading activated anterior regions of the left inferior frontal gyrus, over and above the predicted correlation between neural activity and RT, and to a greater extent than pseudoword relative to word reading. Again, if our RT measure adequately captures processing effort, this result indicates that irregular words engage somewhat different prefrontal mechanisms than pseudowords. We argued that irregular words engage multiple phonological representations that must be selected between, an idea that resonates with recent ideas in the neuroscientific, psycholinguistic, and developmental literature. Future investigations could use multi-voxel pattern analysis techniques to examine whether activation of multiple pronunciations during irregular word reading can be observed in anterior regions of inferior frontal gyrus. If confirmed, this would motivate further research to understand the possible advantages conferred by storing multiple phonological forms.

### Relationship between reading and multiple demand networks

The frontal and parietal regions activated for pseudowords relative to words to some extent overlap with those that have been described by Duncan (2010) as part of the multiple demand (MD) network (along with medial prefrontal and inferior temporal regions). These MD regions are active across a broad range of tasks relative to rest, and are more active for more cognitively challenging tasks. It is this overlap, along with the correlation between activity and RT during reading aloud, that has led some neuroimaging researchers to propose that these regions perform domain general, rather than reading related, processes (Binder et al., 2005, Graves et al., 2010). However, we argue that activation during multiple tasks does not preclude the possibility that these regions also contribute to processes that are integral to reading. Duncan (2010) proposed that frontal and parietal cortices are involved in breaking down a task into components, and in allocating and maintaining attention to the current component, with neurons in these regions rapidly adapting to represent task relevant stimuli and perform task relevant operations (Stokes et al., 2013, Woolgar et al., 2011). We propose that, during reading, these regions are involved in breaking down the visual forms of words into their components (left occipitotemporal/parietal cortices), serially attending to these components in order to retrieve the corresponding verbal codes (left parietal cortex), and assembling these verbal codes into a coherent output (left inferior frontal and precentral gyri). Thus, whilst the contrast [pseudowords − words] does activate the MD network, this is because MD regions are involved in processes that are integral to reading aloud that are more effortful for pseudowords. Frontoparietal networks can therefore be considered both domain general *and* responsible for reading related processes.

### General principles for modelling RT in functional imaging studies

Behavioural studies often use RT as their dependent measure of interest. Neuroimaging studies often examine brain activity for contrasts of interest over and above correlations between activity and RT. To resolve this conflict we proposed a framework that can be used by researchers to predict whether between-condition differences in activity should (or should not) be independent of RT, and described an analysis method that can separate RT associated and non-associated activation differences. According to our framework, a researcher conducting univariate analyses of fMRI data should first determine which (if any) of their contrasts of interest are designed to tap differences in the extent to which stimulus types *engage* particular brain regions, i.e. because a region is thought to represent one stimulus type but not another. The researcher can expect such contrasts to reveal activity that is independent of RT. They should then determine which (if any) of their contrasts are designed to tap differences in the *effort* required to process particular stimulus types, i.e. because a particular brain region represents all stimulus types, but some fit the representations better than others. Such contrasts should reveal activity that is correlated with RT, but is still of interest for the task under investigation. To use this framework it is therefore necessary to have a cognitive theory that specifies the nature of the representations in the processing components of interest, and to assume that different processing components can be mapped onto separate brain processes. Predictions as to whether contrasts should tap engagement or effort can then be explicitly tested by constructing general linear models both with and without RT entered as a covariate, as exemplified by the current study.

## Appendix A Supplementary data

Supplementary Table 1 RT correlations in Model 3. p < .001 whole brain voxel-wise uncorrected, and p < .05 cluster-level FWE corrected. All peaks > 12 mm apart are reported.

## Footnotes

4 We also investigated whether RT showed a nonlinear relationship with BOLD signal by including a quadratic RT regressor as an additional parametric modulator. There was no significant effect of this additional regressor in regions that were active during reading aloud relative to rest. However, the quadratic RT regressor was significantly related to BOLD signal (p < .001 voxel-wise uncorrected and p < .05 FWE cluster corrected) in the right angular gyrus, precuneus, and right middle frontal gyrus, regions that were deactive during reading relative to rest. To investigate this further, we hypothesized that for the majority of trials deactivation was greater for longer RTs, but for some trials participants may have been distracted, resulting in very long RTs but reduced deactivation, as distraction is similar to the resting state. To assess this possibility we reanalysed the data excluding trials with RTs greater than two standard deviations above the mean. In this analysis we no longer observed a significant quadratic relationship between RT and BOLD, supporting our hypothesis. This issue should be investigated further but is beyond the scope of the current paper.

5 We also investigated the possibility that the relationship between RT and BOLD signal differed for words relative to pseudowords, by including an RT-by-condition interaction term as an additional parametric modulator in Model 2. We did the same for Model 3, to determine whether the relationship between RT and BOLD signal differed for regular relative to irregular words. However, there was no significant effect of these interaction terms in either model (even at p < .001 uncorrected), indicating that our initial assumption, that the slope of the relationship governing RT and BOLD is the same across item types (as embodied in Fig. 2), holds for the present dataset.
