# Supplementary material for: Interpreting response time effects in functional imaging studies
Source: Neuroimage. 2014 Oct 1;99:419–33. doi: 10.1016/j.neuroimage.2014.05.073 (PMC4121088; doi:10.1016/j.neuroimage.2014.05.073)
Supplement: Supplementary Table 1 — RT correlations in Model 3. p < .001 whole brain voxel-wise uncorrected, and p < .05 cluster-level FWE corrected. All peaks > 12 mm apart are reported. [file mmc1.docx]

*Supplementary Table 1. RT correlations in Model 3. p < .001 whole brain voxel-wise uncorrected, and p < .05 cluster-level FWE corrected. All peaks > 12mm apart are reported*

| Region | Hemisphere | X | Y | Z | N voxels | Z | p |  |
| --- | --- | --- | --- | --- | --- | --- | --- | --- |
| Positive RT correlations |  |  |  |  |  |  |  |  |
| supplementary motor area | bilateral | 0 | 8 | 56 | 2481 | 5.94 | < .001 |  |
| mid-cingulate |  | -6 | 20 | 38 |  |  |  |  |
| mid-cingulate |  | 10 | 18 | 46 |  |  |  |  |
| supplementary motor area |  | 16 | 10 | 68 |  |  |  |  |
| anterior cingulate |  | 6 | 14 | 28 |  |  |  |  |
| anterior cingulate |  | 0 | 38 | 28 |  |  |  |  |
| precentral gyrus | L | -54 | -2 | 44 | 5042 | 5.86 | < .001 |  |
| superior temporal pole |  | -50 | 8 | 0 |  |  |  |  |
| inferior frontal gyrus(oper) |  | -54 | 12 | 30 |  |  |  |  |
| insula |  | -26 | 22 | 8 |  |  |  |  |
| inferior frontal gyrus(tri) |  | -42 | 12 | 26 |  |  |  |  |
| insula |  | -34 | 10 | 6 |  |  |  |  |
| inferior frontal gyrus(oper) |  | -60 | 8 | 10 |  |  |  |  |
| insula |  | -28 | 24 | -6 |  |  |  |  |
| superior temporal pole |  | -54 | -2 | -8 |  |  |  |  |
| inferior frontal gyrus(tri) |  | -50 | 32 | 8 |  |  |  |  |
| postcentral gyrus |  | -56 | -14 | 22 |  |  |  |  |
| middle temporal gyrus |  | -54 | -16 | -2 |  |  |  |  |
| postcentral gyrus |  | -64 | -4 | 26 |  |  |  |  |
| inferior frontal gyrus(tri) |  | -44 | 32 | 20 |  |  |  |  |
| inferior frontal gyrus(tri) |  | -40 | 32 | 0 |  |  |  |  |
| postcentral gyrus |  | -40 | -16 | 40 |  |  |  |  |
| superior temporal pole |  | -32 | 14 | -20 |  |  |  |  |
| precentral gyrus |  | -40 | -2 | 58 |  |  |  |  |
| insula | R | 36 | 24 | 8 | 2252 | 5.31 | < .001 |  |
| insula |  | 46 | 16 | -2 |  |  |  |  |
| superior temporal pole |  | 60 | -24 | 2 |  |  |  |  |
| superior temporal pole |  | 50 | -30 | -2 |  |  |  |  |
| superior temporal pole |  | 50 | -16 | 4 |  |  |  |  |
| middle temporal gyrus |  | 54 | -40 | 4 |  |  |  |  |
| insula |  | 30 | 18 | -8 |  |  |  |  |
| superior temporal gyrus |  | 60 | -6 | 0 |  |  |  |  |
| superior temporal pole |  | 58 | 6 | -4 |  |  |  |  |
| precentral gyrus | R | 50 | 0 | 44 | 1323 | 5.17 | < .001 |  |
| postcentral gyrus |  | 60 | -2 | 36 |  |  |  |  |
| inferior frontal gyrus(oper) |  | 48 | 12 | 22 |  |  |  |  |
| inferior frontal gyrus(oper) |  | 58 | 20 | 20 |  |  |  |  |
| precentral gyrus |  | 56 | 2 | 24 |  |  |  |  |
| precentral gyrus |  | 44 | 6 | 32 |  |  |  |  |
| rolandic operculum |  | 64 | -4 | 12 |  |  |  |  |

Supplementary Table 1 continued.

| Region | Hemisphere | X | Y | Z | | N voxels | Z | p |  |
| --- | --- | --- | --- | --- | --- | --- | --- | --- | --- |
| superior parietal cortex | L | -22 | -62 | 46 | | 768 | 4.54 | < .001 |  |
| inferior parietal cortex |  | -34 | -48 | 46 |  |  |  |  |  |
| superior occipital cortex |  | -22 | -74 | 36 |  |  |  |  |  |
| inferior parietal cortex |  | -46 | -42 | 48 |  |  |  |  |  |
| inferior parietal cortex |  | -42 | -50 | 60 |  |  |  |  |  |
| supramarginal gyrus |  | -50 | -38 | 34 |  |  |  |  |  |
| inferior temporal gyrus | R | 44 | -58 | -12 |  | 404 | 4.51 | < .01 |  |
| inferior occipital cortex | L | -42 | -66 | -4 |  | 950 | 4.5 | < .001 |  |
| fusiform gyrus |  | -46 | -56 | -14 |  |  |  |  |  |
| inferior temporal gyrus |  | -48 | -44 | -18 |  |  |  |  |  |
| inferior parietal cortex | R | 30 | -50 | 46 |  | 1145 | 4.21 | < .001 |  |
| superior occipital cortex |  | 28 | -66 | 40 |  |  |  |  |  |
| inferior parietal cortex |  | 56 | -36 | 52 |  |  |  |  |  |
| superior parietal cortex |  | 22 | -68 | 52 |  |  |  |  |  |
| supramarginal gyrus |  | 48 | -38 | 40 |  |  |  |  |  |
| inferior parietal cortex |  | 44 | -40 | 52 |  |  |  |  |  |
| Negative RT Correlations |  |  |  |  |  |  |  |  |  |
| medial superior frontal gyrus | bilateral | 10 | -52 | 24 | 2918 | 5.46 | < .001 |  |  |
| precuneus |  | -2 | -56 | 22 |  |  |  |  |  |
| precuneus |  | 0 | -58 | 36 |  |  |  |  |  |
| posterior cingulate |  | -12 | -48 | 32 |  |  |  |  |  |
| calcarine cortex |  | -20 | -56 | 16 |  |  |  |  |  |
| cingulate |  | -2 | -36 | 38 |  |  |  |  |  |
| angular gyrus | R | 54 | -58 | 26 | 455 | 5.02 | < .01 |  |  |
| angular gyrus |  | 46 | -66 | 38 |  |  |  |  |  |
| medial orbitofrontal cortex | bilateral | -4 | 58 | -6 | 883 | 4.54 | < .001 |  |  |
| medial superior frontal gyrus |  | 6 | 56 | 8 |  |  |  |  |  |
| medial orbitofrontal cortex |  | -8 | 46 | -6 |  |  |  |  |  |
| medial orbitofrontal cortex |  | 4 | 42 | -12 |  |  |  |  |  |
| middle frontal gyrus | L | -24 | 32 | 40 | 297 | 4.48 | < .05 |  |  |
| middle frontal gyrus |  | -28 | 16 | 42 |  |  |  |  |  |
| middle frontal gyrus | R | 28 | 22 | 48 | 316 | 4.44 | .01 |  |  |
| superior frontal gyrus |  | 22 | 38 | 42 |  |  |  |  |  |
| angular gyrus |  | -44 | -64 | 42 | 522 | 4.1 | 0.001 |  |  |
| angular gyrus | L | -42 | -62 | 26 |  |  |  |  |  |
